# An Approach to Determining Attribute Weights Based on Integrating Preference Information on Attributes with Decision Matrix

**DOI:** 10.1155/2018/4864517

**Published:** 2018-10-01

**Authors:** Quan Zhang, HongWei Xiu

**Affiliations:** ^1^School of Information Engineering, Shenyang University of Technology, Shenyang, China; ^2^Alumni Association, Shenyang Jianzhu University, Shenyang, China

## Abstract

The interval multiple attribute decision-making problems are studied in this paper, where the preference information on attributes is expressed with preference orderings, linguistic terms, interval numbers, and inequality constraints among partial attribute weights. An approach is proposed to determine the attribute weights based on the preference information on attributes and the interval decision matrix. Firstly, preference orderings, linguistic terms, and interval numbers are normalized and aggregated into the group opinions, based on which an optimization model is set up to calculate the subjective attribute weights by including inequality constraints among partial attribute weights in the model. Then, based on the interval decision matrix, the entropy method is adopted to calculate the objective attribute weights, which is integrated with the subjective weights so that both the subjective preference information and the objective information in the decision matrix are reflected. Finally, an example is used to illustrate the proposed approach.

## 1. Introduction

Multiple attribute decision making (MADM) refers to ranking or selecting the alternatives that are associated with noncommensurate and conflicting attributes [1–3]. In the course of multiple attribute decision making, experts are usually invited to help making decisions by providing their preference information against attributes or alternatives [4, 5]. The preference given by experts belongs to the subjective information [6, 7], which can be used to calculate the attribute weights. Given a decision matrix, the attribute weights decide alternative rankings or the best choice among them [8, 9]. While determining the attribute weights, commonly, there are two types of methods: the methods based on the subjective information [8, 9] (that is called the subjective method in this paper) and those based on the decision matrix (that is called the objective method in this paper).

On the one hand, if only subjective information is used for determining the attribute weights, it would result in prejudice or deviation from the objective facts because it only reflects experts' subjective opinions [6, 10]. On the other hand, the attribute weights can also be determined based on the decision matrix. The attribute values of alternatives in the decision matrix are considered as the objective information [3, 6]. If the attribute weights are determined only based on the decision matrix, they would be meaningless because of neglecting the experts' subjective information [11]. Thus, in the course of determining attribute weights, both the subjective method and the objective method have their limitations, respectively. How to combine these two types of methods to reflect both the experts' subjective preference information and the objective information in decision matrix is a problem worth studying [11–14].

This paper focuses on determining the attribute weights based on experts' subjective preference information and the interval decision matrix. The experts' subjective preference information plays important roles in determining the attribute weights [4, 7]. It is noticed that, in fuzzy or uncertain environment, it is difficult to obtain precise decision information [15]. Especially, when the experts involved have different culture and education backgrounds, they often intend to present their subjective preference information on the attribute weights by means of their most easy ways, for example, preference orderings [16], linguistic terms [3, 13, 17], interval numbers [7], or inequality constraints among the partial attribute weights [18, 19].

Integration of decision information is desirable for solving uncertain MADM problems [20]. Currently, there is seldom research on integrating the decision matrix with the experts' subjective preference information on attribute weights [21]. In [22], the auctioneer provides preference on attribute weights, as well as on attribute values and alternatives. The preference on attribute weights is presented with the form of linear inequalities. In [23], the attribute values in decision matrix are presented with crisp values, fuzzy numbers, interval numbers, and linguistic terms. Two types of subjective attribute weights are provided: multiplicative preference relations and fuzzy preference relations.

However, in fuzzy or uncertain decision making environment, for example, the assessment of international cooperation projects across different countries and regions, the experts are invited from different countries and regions and usually have difficulty in giving precise preference information on the attributes, for example, multiplicative preference relations and fuzzy preference relations. In addition, the experts would like to use easier ways to give their preference information on the attribute weights, such as preference orderings, linguistic terms, interval numbers, and inequality constraints among the attribute weights. Instead of utility values, preference orderings are easy ways for experts to describe the relative importance of attributes when they can only give rankings of the attributes. Linguistic terms are natural ways for experts to present their preference information, which can reduce the burden for them to express their opinions. Interval numbers are also the easy ways for experts to present their preference information when they could not give exact values of the attribute weights. Inequality constraints among the attribute weights usually describe the relative comparisons among partial attributes, for example, one attribute is more important than another one.

It is desirable to deal with preference orderings, linguistic terms, interval numbers, and inequality constraints among the attribute weights in the MADM process since they are the common ways for experts to use when expressing their subjective preference on attributes easily and accurately. Furthermore, it is desirable to determine the attribute weights by integrating the interval decision matrix with experts' preference information on attribute weights in the forms of preference orderings, linguistic terms, interval numbers, and inequality constraints among the attribute weights.

The purpose of this paper is to propose an approach to determining attribute weights based on integrating interval decision matrix and the experts' preference information on attributes in the formats of preference orderings, linguistic terms, interval numbers, and inequality constraints among them. The organization of this paper is as follows: the MADM problem with interval decision matrix and experts' preference information on attributes is described in Section 2.  Section 3 proposes the approach to determining the attribute weights by addressing the normalization and aggregation process for the preference information and setting up an integrating optimization model. In Section 4, an example is used to illustrate the proposed approach. Summary is given in Section 5.

## 2. Problem Descriptions

In order to facilitate describing the MADM problem with interval decision matrix and experts' preference information on attributes in the formats of preference orderings, linguistic terms, interval numbers, and inequality constraints among the attribute weights, the following assumptions and notations are adopted:

let *S*={*S*_1_, *S*_2_, ..., *S*_*m*_} denote a discrete set of *m*(≥2) possible alternatives;

let *C*={*c*_1_, *c*_2_, ..., *c*_*n*_} denote a set of *n*(≥2) attributes;

let *W*=(*w*_1_, *w*_2_, ..., *w*_*n*_) denote the vector of *n*(≥2) attribute weights, where *w*_*j*_ is the weight of attribute *c*_*j*_, while ∑_*j*=1_^*n*^*w*_*j*_=1 and *w*_*j*_ ≥ 0 holds for *j*=1,…, *n*.

Let $\tilde{A}=\left[{\tilde{a}}_{ij}\right]$ denote the interval decision matrix, where, ${\tilde{a}}_{ij}=\left[a_{ij}^{L},a_{ij}^{U}\right]$ is the interval consequence for alternative ${\tilde{q}}_{ij}$ with respect to attribute *c*_*j*_, *B*=[*b*_*ij*_]_*m*×*n*_, *j*=1,…, *n*.

The experts involved are known: let *E* = {*e*_1_, *e*_2_,…, *e*_*K*_}(*k* ≥ 2) denote the set of experts. Different experts express their subjective preference information on attributes in the following formats, i.e., preference orderings, linguistic terms, interval numbers, and inequality constraints among the attribute weights, as stated in Table 1.

The problem left is to propose an approach to determining the attribute weights based on integrating the interval decision matrix and the experts' preference information on attributes in the formats of preference orderings, linguistic terms, interval numbers, and inequality constraints among them, as stated in Table 1.

## 3. The Proposed Approach

### 3.1. Determining Attribute Weights Based on the Experts' Preference Information

As stated in Table 1, in this paper, the experts express their subjective preference information on attributes in the formats of preference orderings, linguistic terms, interval numbers, and inequality constraints among them. In order to determine the attribute weights based on the experts' preference information, normalization and aggregation would be employed. Based on the aggregation results, preference exploitation would be conducted by setting up an optimization model including the inequality constraints among the attribute weights.

In order to facilitate describing the proposed approach, the definition of fuzzy preference relation is given firstly.

*Definition 1*. A fuzzy preference relation on the attributes is a binary fuzzy relation *P* on *C*, where *P* is a mapping *C* × *C*⟶[0,1] and *p*_*jr*_ denotes the preference degree of attribute *c*_*j*_ over *c*_*r*_. It is assumed that *P* is reciprocal, by definition, (i) *p*_*jr*_+*p*_*rj*_=1 and (ii) *p*_*jj*_=− (symbol “−” means that there is no need to give any preference information on attribute *c*_*j*_).

#### 3.1.1. Normalize Experts' Preference Information

To normalize the experts' preference information on attributes in the formats of preference orderings, linguistic terms, and interval numbers, the definition of linguistic terms is presented as follows, as well as the basic linguistic evaluation set is addressed.

*Definition 2*. A linguistic term $\tilde{T}$ on real number set is defined as a triangular fuzzy number (denoted as (*u*, *α*, *β*)), if its membership function $\mu_{\tilde{T}}\left(R^{+}⟶\left[0,1\right]\right)$ is defined as,

(1) $$\begin{array}{c} \mu_{\tilde{T}}\left(x\right)=\left\{\begin{array}{cc} \frac{x-\alpha }{u-\alpha }, & x\in \left[\alpha ,u\right],\\ \frac{x-\beta }{u-\beta }, & x\in \left[u,\beta \right],\\ 0, & \text{otherwise,} \end{array}\right. \end{array}$$

where *α* ≤ *u* ≤ *β*, and *u* is the model value and *α* and *β* stand for the lower value and the upper value of linguistic term $\tilde{T}$, respectively.

A linguistic term set {*t*_0_, *t*_1_,…, *t*_*g*_} is an ordering set, which is composed of a number of linguistic terms with odd number (i.e., *g* + 1 is the odd number), for example, a set of seven terms, i.e., {*t*_0_ = “none,” *t*_1_ = “very poor,” *t*_2_ = “poor” *t*_3_ = “fair,” *t*_4_ = “good,” *t*_5_ = “very good,” amd *t*_6_ = “perfect”}. The following properties of linguistic term set {*t*_0_, *t*_1_,…, *t*_*g*_} are assumed [7, 24]: (1) it is ordered: *t*_*i*_ ≥ *t*_*j*_, if *i* ≥ *j*. The symbol “≥” denotes “better or equal”; (2) there is the negation operator “Neg”: Neg (*t*_*i*_) = *t*_*j*_, such that *j* = *g* − *i*, where *g* + 1 is the number of elements in {*t*_0_, *t*_1_,…, *t*_*g*_}, and the largest term is *t*_*g*_; (3) There is the max operator and the min operator: Max {*t*_*i*_, *t*_*j*_} = *t*_*i*_ and Min {*t*_*i*_, *t*_*j*_} = *t*_*j*_ if *t*_*i*_ ≥ *t*_*j*_.

Since different experts would use different linguistic term sets when expressing their subjective preference information against the attributes, the linguistic term sets would be of different granularity. A basic linguistic evaluation term set would be used for normalizing the linguistic terms with different granularity. In this paper, the basic linguistic evaluation term set TERMSET is defined as {term_0_, term_1_, … , term_*g*_}, where *g* + 1 is the odd number. The membership functions (including the model value *u*_*l*_, the lower value *α*_*l*_, and the upper value *β*_*l*_) of triangular fuzzy numbers *γ*_*l*_=(*u*_*l*_, *α*_*l*_, *β*_*l*_) for the elements in the basic linguistic evaluation set (i.e., TERMSET = {term_0_, term_1_,…, term_*g*_}) are defined as in (2), *l*=0,1,…, *g*.

(2) $$\begin{array}{c} \gamma_{l}=\left\{\begin{array}{cc} \alpha_{0}=0,\\ u_{l}=\frac{l}{g-1}, & 0\leq l\leq g-1,\\ \alpha_{l}=\frac{l-1}{g-1}, & 1\leq l\leq g-1,\\ \beta_{l}=\frac{l+1}{g-1}, & 0\leq l\leq g-2,\\ \beta_{g-1}=1, \end{array}\right. \end{array}$$

where *g* + 1 is the number of terms in the basic linguistic evaluation set TERMSET.

In this paper, {“less important,” “unimportant,” “fair,” “important,” and “very important”} is adopted as the basic linguistic evaluation set TERMSET.

1. Transform preference orderings into fuzzy preference relations

Given preference orderings *O*^*k*^=(*o*_1_^*k*^, *o*_2_^*k*^,…, *o*_*n*_^*k*^) by expert *e*_*k*_(*k*=1,…, *K*), the following steps can be used to transform *O*^*k*^=(*o*_1_^*k*^, *o*_2_^*k*^,…, *o*_*n*_^*k*^) into the fuzzy preference relations on attributes.

*Step 1*. Set up the corresponding intervals for *n* ranking positions, inter_*j*_=[(*n* − *j*/*n*), (*n*+1 − *j*/*n*)], 1 ≤ *j* ≤ *n*.

*Step 2*. Set up the membership functions for the corresponding interval of the ranking positions, i.e.,

(3) $$\begin{array}{c} y\left(j\right)=\left\{\begin{array}{c} 1,\;\frac{n-j}{n}\leq x\leq \frac{n+1-j}{n},\\ 0,\;\text{others}. \end{array}\right. \end{array}$$

Thus, the preference ordering of attribute *c*_*j*_ can be transformed into a subset in [0, 1].

*Step 3*. Based on the interval membership functions corresponding to the ranking positions of attribute *c*_*j*_, the following definition 3 can be used to transform inter_*j*_ into a fuzzy set over the basic linguistic evaluation set TERMSET, *j*=1,…, *n*.

*Definition 3*. Suppose $\tilde{\lambda }$ is an interval and TERMSET={term_0_, term_1_,…, term_*g*_} is the basic linguistic evaluation set, by means of the following mapping, $\tilde{\lambda }$ can be transformed into a fuzzy set over TERMSET,

(4) $$\begin{array}{c} \tau :\tilde{\lambda }⟶F\left(\text{TERMSET}\right). \end{array}$$

Formula (4) can be rewritten as the form of fuzzy set over TERMSET,

(5) $$\begin{array}{c} \tau \left(\tilde{\lambda }\right)=\left\{\left.\left({\text{term}}_{l},\gamma_{l}\right)\right|l\in \left[0,g\right]\right\}, \end{array}$$

where

(6) $$\begin{array}{c} \gamma_{l}=\underset{y}{\max}\min\left\{\mu_{\tilde{\lambda }}\left(y\right),\mu_{{\text{term}}_{l}}\left(y\right)\right\}, \end{array}$$

where $\mu_{\tilde{\lambda }}\left(y\right)$ and *μ*_term_*l*__(*y*) are the membership functions of $\tilde{\lambda }$ and term_*l*_, respectively [24].

Denote *τ*(*o*_*j*_^*k*^)=*F*_*j*_^*k*^(TERMSET) as the fuzzy set over TERMSET, for the ranking position *o*_*j*_^*k*^, *j*=1,…, *n*.

*Step 4*. Transform *F*_*j*_^*k*^(TERMSET) into the fuzzy preference relations on the attributes.

Suppose *F*_*j*_^*k*^(TERMSET)={(term_0_, *γ*_*j*,0_^*k*^), (term_1_, *γ*_*j*,1_^*k*^),…(term_*g*_, *γ*_*j*,*g*_^*k*^)} is the fuzzy set over TERMSET for the ranking position *o*_*j*_^*k*^, *j*=1,…, *n*, which is obtained in Step 3. The following mapping *χ* in (7) can be used to transform *F*_*j*_^*k*^(TERMSET) into a crisp value:

(7) $$\begin{array}{c} \chi :F_{j}^{k}\left(\text{TERMSET}\right)⟶\left[0,g\right], \end{array}$$

where formula (7) is stated as

(8) $$\begin{array}{c} \chi \left(F_{j}^{k}\left(\text{TERMSET}\right)\right)=\frac{\sum_{l=0}^{g}\gamma_{j,l}^{k}\cdot l}{g\sum_{l=0}^{g}\gamma_{j,l}^{k}}. \end{array}$$

Therefore, given the preference orderings *O*^*k*^=(*o*_1_^*k*^, *o*_2_^*k*^,…, *o*_*n*_^*k*^) by expert *e*_*k*_(*k*=1,…, *K*), the fuzzy preference relations between *c*_*j*_ and *c*_*r*_ can be obtained as in the following equation:

(9) $$\begin{array}{c} p_{jr}^{k}=\frac{\chi \left.\left(F_{j}^{k}\text{TERMSET}\right)\right)}{\chi \left(F_{j}^{k}\left(\text{TERMSET}\right)\right)+\chi \left(F_{r}^{k}\left(\text{TERMSET}\right)\right)},\;j,r=1,\ldots ,n. \end{array}$$

(2) Transform linguistic terms into fuzzy preference relations

Suppose expert *e*_*k*_(*k*=1,…, *K*) gives his/her preference against the attributes with a linguistic term vector ling^*k*^=(ling_1_^*k*^,…, ling_*n*_^*k*^), and ling_*j*_^*k*^ is the linguistic term for attribute *c*_*j*_, then the following method is used to transform ling_*j*_^*k*^ into the fuzzy set over TERMSET:

(10) $$\begin{array}{c} \tau \left({\text{ling}}_{j}^{k}\right)=\left.\left({\text{term}}_{l},\gamma_{j,l}^{k}\right)\right|l\in \left[0,g\right],\;j=1,\ldots n, \end{array}$$

where

(11) $$\begin{array}{c} \gamma_{j,l}^{k}=\underset{y}{\max}\min\left\{\mu_{{\text{ling}}_{j}^{k}}\left(y\right),\mu_{{\text{term}}_{l}}\left(y\right)\right\}, \end{array}$$

where *μ*_ling_*j*_^*k*^_(*y*) and *μ*_term_*l*__(*y*) are the membership functions of ling_*j*_^*k*^ and term_*l*_, respectively.

Therefore, given the preference information of linguistic term vector ling^*k*^=(ling_1_^*k*^,…, ling_*n*_^*k*^) from expert *e*_*k*_(*k*=1,…, *K*), the fuzzy preference relation between *c*_*j*_ and *c*_*r*_ is obtained as follows:

(12) $$\begin{array}{c} p_{jr}^{k}=\frac{\chi \left(\tau \left({\text{ling}}_{j}^{k}\right)\right)}{\chi \left(\tau \left({\text{ling}}_{j}^{k}\right)\right)+\chi \left(\tau \left({\text{ling}}_{r}^{k}\right)\right)}\;j,r=1,\ldots ,n, \end{array}$$

where

(13) $$\begin{array}{c} \chi \left(\tau \left({\text{ling}}_{j}^{k}\right)\right)=\frac{\sum_{l=0}^{g}\gamma_{j,l}^{k}\cdot l}{\sum_{l=0}^{g}\gamma_{j,l}^{k}}. \end{array}$$

(3) Transform interval numbers into fuzzy preference relations

Suppose expert *e*_*k*_(*k*=1,…, *K*) gives his/her preference information on the attributes in the format of interval numbers, for example, ineral_*j*_^*k*^=[ineral_*j*_^*kL*^, ineral_*j*_^*kU*^] is for attribute *c*_*j*_ and ineral_*r*_^*k*^=[ineral_*r*_^*kL*^, ineral_*r*_^*kU*^] is for attribute *c*_*r*_, then, the fuzzy preference relations between *c*_*j*_ and *c*_*r*_ is obtained as follows [25]:

(14) $$\begin{array}{c} p_{jr}^{k}=\frac{\max\left\{0,\text{len}\left({\text{ineral}}_{j}^{k}\right)+\text{len}\left({\text{ineral}}_{r}^{k}\right)-\max\left({\text{ineral}}_{r}^{kU}-{\text{ineral}}_{j}^{kL},0\right)\right\}}{\text{len}\left({\text{ineral}}_{j}^{k}\right)+\text{len}\left({\text{ineral}}_{r}^{k}\right)},\;j,r=1,\ldots ,n, \end{array}$$

where len(ineral_*j*_^*k*^) and len(ineral_*r*_^*k*^) are the lengths of ineral_*j*_^*k*^ and ineral_*r*_^*k*^, respectively.

(15a) $$\begin{array}{c} \text{len}\left({\text{ineral}}_{j}^{k}\right)={\text{ineral}}_{j}^{kU}-{\text{ineral}}_{j}^{kL}, \end{array}$$

(15b) $$\begin{array}{c} \text{len}\left({\text{ineral}}_{r}^{k}\right)={\text{ineral}}_{r}^{kU}-{\text{ineral}}_{r}^{kL}. \end{array}$$

#### 3.1.2. Aggregate Experts' Preference Information

After normalizing the experts' subjective preference information on the attributes in 3.1.1, their preference information are transformed into the fuzzy preference relations, respectively, and the next step is to aggregate these resulting fuzzy preference relations. In this paper, the “simple additive weighting method” is used to aggregate these resulting fuzzy preference relations from the experts, i.e.,

(16) $$\begin{array}{c} p_{jr}=\overset{K}{\underset{k=1}{\sum }}\lambda_{k}\cdot p_{jr}^{k},\;k=1,\ldots ,K,j,r=1,\ldots ,n, \end{array}$$

where *p*_*jr*_^*k*^ is the fuzzy preference relations between attributes *c*_*j*_ and *c*_*r*_, which is derived from expert *e*_*k*_'s preference information, *k*=1,…, *K*, *j*, *r*=1,…, *n*. *λ*_*k*_ is the weight of expert *e*_*k*_.

Denote the group fuzzy preference relations as *P*=(*p*_*jr*_)_*n*×*n*_, where *p*_*jr*_ is obtained in (16).

#### 3.1.3. Determine Attribute Weights Based on the Experts' Preference Information

Given the group fuzzy preference relations *P*=(*p*_*jr*_)_*n*×*n*_ obtained in (16), *p*_*jr*_ should be as close as possible with *w*_*j*_/(*w*_*j*_+*w*_*r*_). It is noticed that *p*_*jr*_+*p*_*rj*_=1, and therefore, the following optimization model can be set up to determine the attribute weights based on the group fuzzy preference relations *P*=(*p*_*jr*_)_*n*×*n*_:

(17a) $$\begin{array}{c} \min Z=\overset{n}{\underset{j=1}{\sum }}\overset{n}{\underset{r=1}{\sum }}{\left(p_{jr}w_{r}-p_{rj}w_{j}\right)}^{2}, \end{array}$$

such that

(17b) $$\begin{array}{c} \overset{n}{\underset{j=1}{\sum }}w_{j}=1, \end{array}$$

(17c) $$\begin{array}{c} w_{j}>0,\;j=1,\ldots ,n. \end{array}$$

If the inequality constraints among attributes are considered, for example, *w*_2_ > *w*_3_, model (17a)–(17c) would be modified into the following one:

(18a) $$\begin{array}{c} \min Z=\overset{n}{\underset{j=1}{\sum }}\overset{n}{\underset{r=1}{\sum }}{\left(p_{jr}w_{r}-p_{rj}w_{j}\right)}^{2}, \end{array}$$

such that

(18b) $$\begin{array}{c} \overset{n}{\underset{j=1}{\sum }}w_{j}=1, \end{array}$$

(18c) $$\begin{array}{c} w_{2}>w_{3}, \end{array}$$

(18d) $$\begin{array}{c} w_{j}\geq 0,\;j=1,\ldots ,n, \end{array}$$

where formula (18c) denotes that attribute *c*_2_ is more important than *c*_3_ (i.e., *w*_2_ > *w*_3_).

Models (18a)–(18d) can be solved by means of Matlab Toolbox. Denote the attribute weight vector obtained by solving Models (18a)–(18d) as *w*′=(*w*_1_′, *w*_2_′,…, *w*_*n*_′), which is derived from the experts' subjective preference information in different formats as stated above.

### 3.2. Determine Attribute Weights Based on the Interval Decision Matrix

Given the interval decision matrix $\tilde{A}={\left[{\tilde{a}}_{ij}\right]}_{m\times n}$, suppose it has been normalized into the beneficial and dimensionless one $\tilde{Q}={\left[{\tilde{q}}_{ij}\right]}_{m\times n}$ [26]. $\tilde{Q}={\left[{\tilde{q}}_{ij}\right]}_{m\times n}$ can be transformed into a single-point one by means of the following operations:

*Definition 4*. Given the beneficial and dimensionless interval decision matrix $\tilde{Q}={\left[{\tilde{q}}_{ij}\right]}_{m\times n}$ (${\tilde{q}}_{ij}=\left[q_{ij}^{L},q_{ij}^{U}\right]$), the base interval for attribute *c*_*j*_ is defined as

(19) $$\begin{array}{c} {\text{pos}}_{j}^{+}=\left[{\text{pos}}_{j}^{+L},{\text{pos}}_{j}^{+U}\right],j=1,\ldots ,n, \end{array}$$

where

(20) $$\begin{array}{c} {\text{pos}}_{j}^{+L}=\underset{1\leq i\leq m}{\min}\left\{q_{ij}^{L}\right\},j=1,\ldots ,n, \end{array}$$

(21) $$\begin{array}{c} {\text{pos}}_{j}^{+U}=\underset{1\leq i\leq m}{\max}\left\{q_{ij}^{U}\right\},j=1,\ldots ,n. \end{array}$$

*Definition 5*. Given the beneficial and dimensionless interval decision matrix $\tilde{Q}={\left[{\tilde{q}}_{ij}\right]}_{m\times n}$, the superiority degree of attribute value ${\overset{˜}{q}}_{ij}$ to the base interval pos_*j*_^+^=[pos_*j*_^+*L*^, pos_*j*_^+*U*^] for attribute *c*_*j*_ is defined as

(22) $$\begin{array}{c} b_{ij}=\frac{q_{ij}^{L}-{\text{pos}}_{j}^{+L}}{{\text{pos}}_{j}^{+U}-{\text{pos}}_{j}^{+L}}+0.5\frac{q_{ij}^{U}-q_{ij}^{L}}{{\text{pos}}_{j}^{+U}-{\text{pos}}_{j}^{+L}},\;i=1,\ldots ,m,j=1,\ldots ,n, \end{array}$$

where pos_*j*_^+*L*^ and pos_*j*_^+*U*^ are defined in (20) and (21), respectively, *j*=1,…, *n*.

Thus, by means of formula (22), the interval decision matrix $\overset{˜}{Q}={\left[{\overset{˜}{q}}_{ij}\right]}_{m\times n}$ is transformed into a single-point one, denoted as *B*=[*b*_*ij*_]_*m*×*n*_. Based on *B*=[*b*_*ij*_]_*m*×*n*_, entropy method can be used to calculate the attribute weight vector [9, 27], denoted as *w*^″^=(*w*_1_^″^, *w*_2_^″^,…, *w*_*n*_^″^).

### 3.3. Determine Attribute Weights Based on Integrating Experts' Subjective Preference Information with Decision Matrix

In order to determine the attribute weights that reflect both the experts' subjective preference information on attributes and the decision matrix, the following integration method is adopted:

(23) $$\begin{array}{c} w=\eta^{\prime }w^{\prime }+\eta^{\prime \prime }w^{\prime \prime }, \end{array}$$

where *w*′ is the attribute weight vector that is derived from the experts' subjective preference information in Section 3.1, and *w*^″^ is the attribute weight vector that is derived from the interval decision matrix in Section 3.2. *η*′ and *η*^″^ denote the relative importance of the experts' subjective preference information and the interval decision matrix, respectively.

*Definition 6*. Given the decision matrix *B*=[*b*_*ij*_]_*m*×*n*_, the positive ideal point for attribute *c*_*j*_ is defined as

(24) $$\begin{array}{c} b_{j}^{+}=\underset{1\leq i\leq m}{\max}\left\{b_{ij}\right\},\;j=1,\ldots ,n. \end{array}$$

Therefore, based on definition 6, the positive ideal alternative *S*^+^ is obtained as (*b*_1_^+^, *b*_2_^+^,…, *b*_*n*_^+^). In addition, based on decision matrix *B*=[*b*_*ij*_]_*m*×*n*_, the weighted sum of the deviations of alternative ${\overset{˜}{q}}_{ij}$ from the positive ideal alternative *S*^+^ is obtained as

(25) $$\begin{array}{c} r_{i}^{+}=\overset{n}{\underset{j=1}{\sum }}w_{j}\left(b_{j}^{+}-b_{ij}\right),\;i=1,\ldots ,m. \end{array}$$

It is obvious that the smaller the deviation from the positive ideal alternative *S*^+^ is, the better the alternative is. Thus, the following optimization model is set up to determine the attribute weights:

(26) $$\begin{array}{c} \min\;r^{+}=\left(r_{1}^{+},r_{2}^{+},\ldots r_{m}^{+}\right). \end{array}$$

Actually, model (26) is a multiobjective optimization model. Since there is no preference against the alternatives, in other words, the alternatives compete fairly, therefore, model (26) can be transformed into a single-objective one:

(27a) $$\begin{array}{c} \min\;Z^{+}=\overset{m}{\underset{i=1}{\sum }}\overset{n}{\underset{j=1}{\sum }}\left(b_{j}^{+}-b_{ij}\right)\left(\eta^{\prime }w^{\prime }+\eta^{\prime \prime }w^{\prime \prime }\right), \end{array}$$

such that

(27b) $$\begin{array}{c} {\eta^{\prime }}^{2}+{\eta^{\prime \prime }}^{2}=1, \end{array}$$

(27c) $$\begin{array}{c} 0\leq \eta^{\prime }\leq 1,0\leq \eta^{\prime \prime }\leq 1. \end{array}$$

Due to the limit of space, the process of solving models (27a)–(27c) is omitted. Denote the optimal solutions to models (27a)–(27c) as:

(28) $$\begin{array}{c} {\eta^{\prime }}^{*}=\frac{\sum_{i=1}^{m}\sum_{j=1}^{n}\left(b_{j}^{+}-b_{ij}\right)w_{j}^{\prime }}{\sqrt{{\left[\sum_{i=1}^{m}\sum_{j=1}^{n}\left(b_{j}^{+}-b_{ij}\right)w_{j}^{\prime }\right]}^{2}+{\left[\sum_{i=1}^{m}\sum_{j=1}^{n}\left(b_{j}^{+}-b_{ij}\right)w_{j}^{\prime \prime }\right]}^{2}}}, \end{array}$$

(29) $$\begin{array}{c} {\eta^{\prime \prime }}^{*}=\frac{\sum_{i=1}^{m}\sum_{j=1}^{n}\left(b_{j}^{+}-b_{ij}\right)w_{j}^{\prime \prime }}{\sqrt{{\left[\sum_{i=1}^{m}\sum_{j=1}^{n}\left(b_{j}^{+}-b_{ij}\right)w_{j}^{\prime }\right]}^{2}+{\left[\sum_{i=1}^{m}\sum_{j=1}^{n}\left(b_{j}^{+}-b_{ij}\right)w_{j}^{\prime \prime }\right]}^{2}}}. \end{array}$$

Normalize the solutions in (28) and (29) so that their sum is equal to 1 and the attribute weight vector *w*^*∗*^ can be obtained as:

(30) $$\begin{array}{c} w^{*}={\eta^{\prime }}^{*}w^{\prime }+{\eta^{\prime \prime }}^{*}w^{\prime \prime }. \end{array}$$

## 4. Illustrations

One investment company intends to evaluate five alternatives (*S*_1_, *S*_2_, *S*_3_, *S*_4_, and *S*_*5*_) [26]. The attributes adopted are investment amount *c*_1_, expected net present value *c*_2_, risk profitability value *c*_3_, and risk loss value *c*_4_. The attribute values of the alternatives are all interval numbers, as stated in Table 2 [26].

Among the four attributes, the expected net present value *c*_2_ and risk profitability value *c*_3_ are for benefits, as investment amount *c*_1_ and risk loss value *c*_4_ are for costs. Four experts are invited for evaluating the alternatives by providing their preference information against the attributes, as stated in Table 1.

Firstly, normalize the experts' subjective preference information in Table 1 and aggregate them into the group fuzzy preference relations on the attributes as follows:

(31) $$\begin{array}{c} p=\left(\begin{array}{cccc} 0.5000 & 0.3833 & 0.6722 & 0.2587\\ 0.6167 & 0.5000 & 0.7944 & 0.3770\\ 0.3278 & 0.2056 & 0.5000 & 0.1250\\ 0.7413 & 0.6247 & 0.8750 & 0.5000 \end{array}\right). \end{array}$$

Secondly, consider the inequality constraints among attributes *c*_2_ and *c*_3_, i.e., *w*_2_ > *w*_3_ in models (18a)–(18d). Then, based on the experts' subjective preference information, the attribute weight vector can be obtained as *w*′ = (0.2237, 0.2101, 0.3126, 0.2536).

Then, normalize the attribute values in the interval decision matrix into dimensionless ones, and the results are stated in Table 3 [26].

Furthermore, the normalized interval decision matrix is transformed into a single-point one *B*=[*b*_*ij*_]_*m*×*n*_ as follows:

(32) $$\begin{array}{c} B=\left(\begin{array}{cccc} \text{0}.6843 & \text{0}.3900 & \text{0}.4684 & \text{0}.5867\\ \text{0}.1334 & \text{0}.6814 & \text{0}.6397 & \text{0}.1653\\ \text{0}.7421 & \text{0}.3900 & \text{0}.2972 & \text{0}.5867\\ \text{0}.1924 & \text{0}.5358 & \text{0}.6397 & \text{0}.1817\\ \text{0}.4975 & \text{0}.1593 & \text{0}.1957 & \text{0}.4134 \end{array}\right). \end{array}$$

Based on decision matrix *B*, by means of entropy method, the attribute weight vector can be obtained as *w*^″^ = (0.3637, 0.1891, 0.1831, 0.2640).

In addition, by means of solving models (27a)–(27c), the relative importances of the experts' subjective preference information and the interval decision matrix are obtained as follows: *η*′^*∗*^ = 0.6874 and *η*^″^^*∗*^ = 0.7263, respectively. After normalizing *η*′^*∗*^ and *η*^″^^*∗*^ into 0.4862 and 0.5138, the comprehensive attribute weight vector can be obtained as *w*^*∗*^ = (0.2956, 0.1993, 0.2461, 0.2589).

## 5. Conclusions

This paper proposes an approach to determining the attribute weights by integrating the interval decision matrix with experts' subjective preference information on attributes in the formats of preference orderings, linguistic terms, interval numbers, and inequality constraints among partial attributes. Preference normalization and aggregation are conducted firstly. Based on the obtained group fuzzy preference relation on the attributes, the optimization models (18a)–(18d) are set up to calculate the subjective weights, while satisfying the inequality constraints among partial attributes. The objective attribute weights are obtained by using entropy method after normalizing the interval decision matrix. The subjective weights and the objective weights are integrated in the optimization models (27a)–(27c) in order to calculate the relative importance of the subjective information and the objective information.

The attribute weights, obtained in the integration models (27a)–(27c), take into account experts' subjective preference information and the objective information of the decision matrix so that they are more reasonable and credible. In addition, this paper enables experts to express their preference information in the easiest ways and accurately, especially in fuzzy or uncertain decision environment. Compared with the current research, the proposed approach has more universal significance and practical application prospect.

## Figures and Tables

**Table 1:** Experts' subjective preference information on attributes.

	*c*_1_	*c*_2_	*c*_3_	*c*_4_
*e*_1_(preference orderings)	3	2	4	1
*e*_2_ (linguistic terms)	Fair	Important	Unimportant	Very important
*e*_3_ (interval numbers)	[0.10, 0.30]	[0.15, 0.35]	[0.15, 0.20]	[0.25, 0.40]
*e*_4_(inequality constraints among attribute weights)	*w*_2_ > *w*_3_			

**Table 2:** The interval attribute values in the decision matrix [22].

	*C*_1_	*C*_2_	*C*_3_	*C*_4_
*S*_1_	[5, 7]	[4, 6]	[4, 6]	[0.4, 0.8]
*S*_2_	[10, 12]	[6, 8]	[5, 7]	[1, 2]
*S*_3_	[5, 6]	[4, 6]	[3, 5]	[0.4, 0.8]
*S*_4_	[9, 11]	[5, 7]	[5, 7]	[1, 1.5]
*S*_*5*_	[6, 8]	[3, 4]	[3, 4]	[0.5, 1]

**Table 3:** The normalized attribute values in the interval decision matrix.

	*C*_1_	*C*_2_	*C*_3_	*C*_4_
*S*_1_	[0.1837, 0.3285]	[0.1290, 0.2727]	[0.1379, 0.3000]	[0.1389, 0.5357]
*S*_2_	[0.1071, 0.1643]	[0.1936, 0.3636]	[0.1724, 0.3500]	[0.0556, 0.2143]
*S*_3_	[0.2143, 0.3285]	[0.1290, 0.2727]	[0.1035, 0.2500]	[0.1389, 0.5357]
*S*_4_	[0.1169, 0.1825]	[0.1613, 0.3182]	[0.1724, 0.3500]	[0.0714, 0.2143]
*S*_*5*_	[0.1607, 0.2738]	[0.0968, 0.1818]	[0.1035, 0.2000]	[0.1111, 0.4286]

## Data Availability

The data used to support the findings of this study are available from the corresponding author upon request.
